# *Lactobacillus reuteri* Reduces the Severity of Experimental Autoimmune Encephalomyelitis in Mice by Modulating Gut Microbiota

**DOI:** 10.3389/fimmu.2019.00385

**Published:** 2019-03-07

**Authors:** Baokun He, Thomas K. Hoang, Xiangjun Tian, Christopher M. Taylor, Eugene Blanchard, Meng Luo, Meenakshi B. Bhattacharjee, Jasmin Freeborn, Sinyoung Park, Jacob Couturier, John William Lindsey, Dat Q. Tran, Jon Marc Rhoads, Yuying Liu

**Affiliations:** ^1^Division of Gastroenterology, Departments of Pediatrics, The University of Texas Health Science Center at Houston-McGovern Medical School, Houston, TX, United States; ^2^Department of Bioinformatics & Computational Biology, The University of Texas MD Anderson Cancer Center, Houston, TX, United States; ^3^Department of Microbiology, Immunology & Parasitology, Louisiana State University, School of Medicine, New Orleans, LA, United States; ^4^Pathology and Laboratory Medicine, University of Texas Health Science Center at Houston-McGovern Medical School, Houston, TX, United States; ^5^Internal Medicine, Division of Infectious Diseases, The University of Texas Health Science Center at Houston-McGovern Medical School, Houston, TX, United States; ^6^Neurology, The University of Texas Health Science Center at Houston-McGovern Medical School, Houston, TX, United States

**Keywords:** experimental autoimmune encephalomyelitis, *Lactobacillus reuteri*, T_H_1/T_H_17 cells, IFN-γ/IL-17, gut microbiota

## Abstract

The gut microbiome plays an important role in immune function and has been implicated in multiple sclerosis (MS). However, how and if the modulation of microbiota can prevent or treat MS remain largely unknown. In this study, we showed that probiotic *Lactobacillus reuteri* DSM 17938 (*L. reuteri*) ameliorated the development of murine experimental autoimmune encephalomyelitis (EAE), a widely used animal model of MS, a model which is primarily mediated by T_H_17 and T_H_1 cells. We discovered that *L. reuteri* treatment reduced T_H_1/T_H_17 cells and their associated cytokines IFN-γ/IL-17 in EAE mice. We also showed that the loss of diversity of gut microbiota induced by EAE was largely restored by *L. reuteri* treatment. Taxonomy-based analysis of gut microbiota showed that three “beneficial” genera *Bifidobacterium, Prevotella*, and *Lactobacillus* were negatively correlated with EAE clinical severity, whereas the genera *Anaeroplasma, Rikenellaceae*, and *Clostridium* were positively correlated with disease severity. Notably, *L. reuteri* treatment coordinately altered the relative abundance of these EAE-associated taxa. In conclusion, probiotic *L. reuteri* changed gut microbiota to modulate immune responses in EAE, making it a novel candidate in future studies to modify the severity of MS.

## Introduction

Multiple sclerosis (MS) is an autoimmune disease of the central nervous system (CNS) which causes neurological disability in up to two million young adults worldwide (1, 2). The key pathological features of MS include axonal loss, demyelination, gliosis, and a progressive inflammatory reaction involving both the adaptive and the innate immune system (3, 4). During the course of MS, activated autoreactive T cells have been proposed to differentiate into CD4^+^ T cells characterized by the production of interferon-γ (IFN-γ) by [T helper 1 (T_H_1) cells] and/or interleukin (IL)-17 by IL-17-producing (T_H_17) cells. These T cells successively induce inflammatory lesions distributed throughout the CNS (2). Infiltration of T_H_1 and T_H_17 CD4^+^ T cells into the CNS is considered an important contributor to the immunopathogenesis of MS (5).

Interestingly, growing evidence from both rodent and human studies suggests that the gut microbiota contributes to the pathogenesis of MS (6–9). In human with MS, an abnormal profile of gut microbiota (dysbiosis) may be one of the several factors involved in the pathogenesis and progression of MS (7–9). In a rodent model of MS, experimental autoimmune encephalomyelitis (EAE), investigative groups have shown that an alteration of the gut microbiota plays a critical role in the pathogenesis of the disease. Supporting this postulate, two studies showed that oral antibiotic administration reduced the severity of EAE (10, 11). Moreover, two studies revealed that germ-free mice were resistant to the development of EAE (6, 12). However, whether remodeling intestinal microbiota can improve EAE symptoms and markers of brain and systemic inflammation remains unknown.

Our previous studies showed that *Lactobacillus reuteri* DSM 17938 (*L. reuteri*) can modulate microbiota and inhibit autoimmunity in an autoimmune disease caused by regulatory T cell (Treg) deficiency (13). In this study, we investigated the effect of *L. reuteri* on EAE and EAE-associated gut microbiota.

## Materials and Methods

### Animals

Female wild-type (WT) C57BL/6 (10 weeks-old) mice were purchased from Jackson Laboratories and allowed to acclimatize for 2–3 weeks before experimentation. The mice were housed in groups in polycarbonate cages with free access to a standard diet and water in the specific pathogen free (SPF) animal facility at The University of Texas Health Science Center at Houston. This study was carried out in accordance with the recommendations of the Guide for the Care and Use of Laboratory Animals (NIH) and The Institutional Animal Care and Use Committee (IACUC). The protocol was approved by the IACUC (protocol numbers: AWC-15-0051 and AWC-18-0051).

### Induction and Assessment of EAE

EAE was induced in female mice by using the Hooke Kit™ MOG_35−55_/CFA Emulsion PTX kit (Hooke Laboratories), according to the manufacturer's protocol. In brief, mice were immunized with an emulsion of myelin oligodendrocyte glycoprotein peptide (MOG_35−55_) in complete Freund's adjuvant (CFA) by subcutaneous injection into two different sites on each hind flank, followed by intraperitoneal administration of pertussis toxin (PTX) in phosphate-buffered saline (PBS) after 2 h MOG_35−55_ immunization. Then PTX was given again on the following day (14). The mice were examined for clinical signs of EAE in a blinded fashion daily from 7 to 20 days after immunization. EAE was scored on scale 0 to 5 (15). Specifically, we assigned Score 0, no obvious changes in motor function compared to normal control mice; Score 1, limp tail; Score 2, limp tail, and weakness of hind legs; Score 3, limp tail and complete paralysis of hind legs; Score 4, limp tail, complete hind leg and partial front leg paralysis, and Score 5, spontaneously rolling in the cage. We assigned mice “in-between” scores (i.e., 0.5, 1.5, 2.5, 3.5) when the clinical picture best fit between two defined scores.

### *L. reuteri* Treatment of EAE Mice

*L. reuteri*, originally isolated from human breast milk, was provided by BioGaia AB (Stockholm, Sweden) and cultured anaerobically in De Man, Rogosa Sharpe (MRS, Fisher Scientific, Pittsburgh, PA) media as described previously (16). Each mouse was assigned to either (a) MRS media as a control (EAE), or (b) *L. reuteri* (EAE+LR), as well as normal control group (Ctrl). On the day 0, mice were given by gavage MRS (100 μl) or *L. reuteri* [10^8^ colony-forming unit (CFU), 100 μl] followed immediately by the injections of MOG_35−55_ in CFA, then PTX after 2 h of MOG_35−55_ injections. On the day 1, EAE mice were given second ip injection of PTX solution. Before the onset of EAE, MRS or *L. reuteri* were given by gavage, daily, starting from day 0 to day 20 after immunization. We observed the mice daily to evaluate the clinical EAE scores. At the end of the experiments on day 20, blood, spleen, and spinal cord, and colonic contents of each mouse were collected. All samples were analyzed at day 20 post-immunization unless specifically indicated.

### Stool Microbial Community Analysis

Colonic contents from Ctrl, EAE, and EAE+LR mice were collected, immediately frozen, and stored at −80°C. The Louisiana State University School of Medicine Microbial Genomics Resource Group (http://metagenomics.lsuhsc.edu) performed sequencing and bioinformatics analysis. Genomic DNA extraction from colonic contents was performed using QIAamp Fast DNA Stool Mini Kit (Qiagen, Germantown, MD) and microbiome 16S rDNA sequencing was performed as previously described (17). The 16S ribosomal DNA hypervariable regions V4 were PCR-amplified using primers V4F GTGCCAGCMGCCGCGGTAA and V4R GGACTACHVGGGTWTCTAAT with Illumina adaptors and molecular barcodes to produce amplicons. Samples were sequenced on Illumina MiSeq (Illumina, San Diego, CA) using V4 sequencing kit. The paired forward and reverse-reads—passed quality control were merged, and then mapped to the SLIVA database to construct OTUs at 97% identity through the UPARSE pipeline (drive5, Tiburon, California) (18). Relative abundance of each OTU was examined at phylum, class, order, family, genus, and species levels. Bacterial alpha and beta diversity metrics, as well as, taxonomic community assessments were produced using QIIME 1.8 (open source, www.qiime.org) (19). The composition of stool microbiota was further analyzed as previously described (20, 21). A systematic search for the genera that correlated with the clinical score was performed by using Spearman's rank correlation coefficient. Random Forest analysis of gut microbiota was performed using the R (http://cran.r-project.org/) package Random Forest (22).

### Histopathology

**Figure 1:**
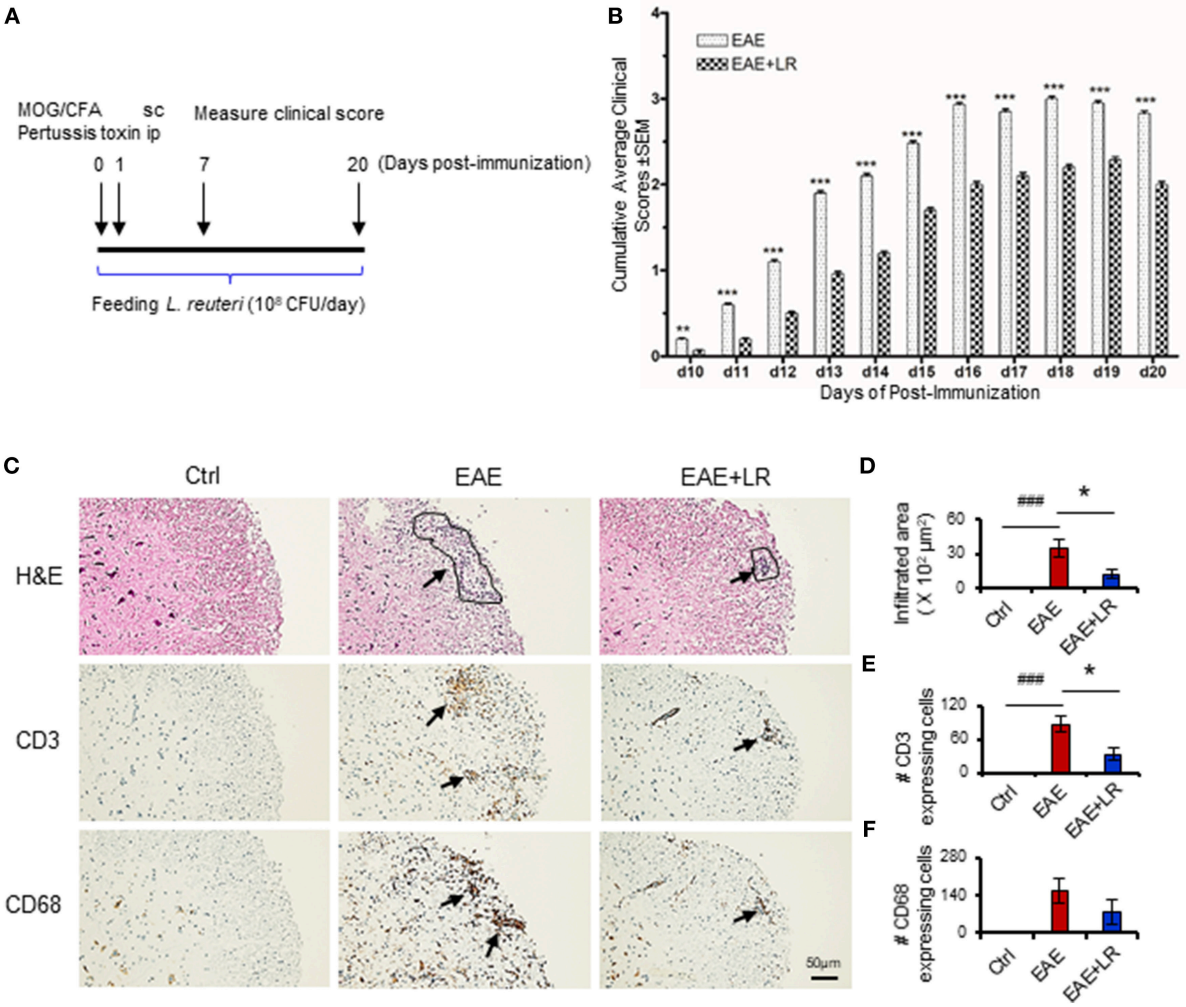
*Lactobacillus reuteri* ameliorates autoimmune disease in EAE mice. **(A)** Scheme of the experimental timeline for administering *L. reuteri* to EAE mice and measuring clinical scores. **(B)** Clinical scores of C57BL/6J mice with EAE to compare with administered MRS and *L. reuteri* (*n* = 37–40 mice per group). **(C)** The representative images of H&E staining, CD3 staining and CD68 staining of spinal cord slides from Ctrl, EAE, and EAE+LR mice (*n* = 10 mice per group). Arrows indicate immune cell infiltration, defined as encircled areas in H&E staining of EAE and EAE+LR specimens. **(D)** The average areas of inflammatory infiltration among group comparisons are shown (see Materials and Methods). **(E)** The average numbers of CD3^+^ cell count per defined area of inflammatory infiltration among group comparisons (see Materials and Methods). **(F)** The average numbers of CD68^+^ cell count per defined area of inflammatory infiltration among group comparisons (see Materials and Methods). Data are presented as mean ± SEM. ^*^p < 0.05, ^**^p < 0.01, ^***^p < 0.001. EAE+LR vs. EAE. ^###^*p* < 0.001. EAE vs. Ctrl.

The spinal cord was fixed and stained with hematoxylin and eosin (H&E), CD3 antibody (Agilent, Denmark) and CD68 (Abcam, Cambridge, MA) antibody for histological evaluation by Histology Laboratory of the Department of Pathology and Laboratory Medicine of UT Health Science Center at Houston. Histological quantification was performed by using Image J software (NIH). We measured the area of inflammatory infiltration as indicated in Figure 1C (H&E staining), three areas of lymphocyte infiltration (if presented) from one section slide, with a total of 2–3 sections for one mouse (from one spinal cord); we measured 10 mice in each group. Finally, we calculated the mean of the areas of lymphocyte infiltration (Figure 1D). For CD3 or CD68 expressing cells, we counted CD3 or CD68 staining positive cells in two fields of high power inside the areas of lymphocyte infiltration, with a total of 2–3 sections from each spinal cord. We measured specimens from 10 mice in each group, finally calculating the mean numbers of CD3 or CD68 expressing cells and their standard deviations.

### *In vitro* Cell Preparation and Stimulation for Flow Cytometry Analysis

Peripheral blood mononuclear cells (PBMCs) were isolated from whole blood by Ficoll-Paque (GE Healthcare, Chicago, IL). Single-cell suspensions from the spleen were prepared by gently fragmenting and filtering the tissues through 40 μm cell strainers (BD Bioscience, San Jose, CA) into MACS buffer (1x PBS, 0.5% bovine BSA and 2 mM EDTA). For *in vitro* stimulation, studies of PBMCs and splenic lymphocytes isolated from each mouse were performed in triplicate, with a total of *n* = 10 mice per group. Briefly, cells were stimulated with 50 ng/mL of phorbol 12-myristate 13-acetate (PMA) and 1 μg/mL of ionomycin in the presence of brefeldin A (5 μ/mL) for 4 h to analyze IFN-γ-producing (T_H_1) and IL-17-producing (T_H_17) CD4^+^ T cells by flow cytometry.

In another set of experiments, splenocytes isolated from mice at day 12 post-immunication were stimulated with 10 μM of MOG_35−55_ for 2 days to analyze cell proliferation.

### *In vitro* Cell Proliferation Assay

Colorimetric measurement for cell proliferation by using the tetrazolium dye, 2,3-bis (2-methoxy-4-nitro-5-sulfophenyl)-5-[(phenylamino) carbonyl]-2H-tetrazolium hydroxide (XTT) was performed according to the manufacturers protocol (TACS^TM^ XTT Cell Proliferation/Viability Assay, R&D Systems, Inc.,) (23). XTT reduction into formazan, a colored compound, by mitochondrial dehydrogenases is considered proportional to cell number and thus related to cell proliferation after incubation time. After 2 days of 10 μM of MOG_35−55_ stimulation, 100 μL of cells were incubated with 50 μL of a mixture of XTT and XTT activator followed by incubation of the plates at 37°C in an atmosphere containing 5% CO2 for 4 h. We measured the absorbance at OD450 nm. We calculated the percentage of proliferation as follows: [(OD_+MOG_-OD_−MOG)_ /OD_−MOG]_ × 100.

### Staining Cells for Flow Cytometry Analysis

For evaluation of T_H_1 and T_H_17 cells, cells were surface-stained by fluorescein-labeled-CD4. Intracellular staining was performed with a fixation/permeabilization kit, according to the manufacturer's protocol (eBioscience/ThermoFisher, Waltham, MA), and stained with IFN-γ (T_H_1) and IL-17 (T_H_17) (BioLegend, San Diego, CA), respectively. The data from all samples were acquired on Gallios flow cytometer (Beckman-Coulter) and analyzed using FlowJo software (FlowJO, LLC) to obtain the percentage of each T cell subsets. The total splenocytes of whole spleen and PBMCs per volume were counted and the absolute numbers of each T cell subsets were calculated, expressed as # of cells per spleen or # of cells/μL in blood.

### Plasma Cytokine Assays

Plasma cytokine levels of IFN-γ and IL-17 were assessed using a mouse multi-spot proinflammatory panel kit from Meso Scale Discovery (MSD, Rockville, MD), according to the manufacturer's protocol. Signals were captured and calculated by Imager 2400 (MSD). The levels of cytokines were expressed as picogram/milliliter (pg/mL).

### Statistical Analysis

Data are presented as mean ± SEM. Statistical significance was determined by one-way ANOVA corrected for multiple comparisons with Tukey and Dunnett's posttests, or two-way ANOVA for multiple comparisons with a Bonferroni test. We used Fisher's Exact Test to compare the incidence of EAE between groups. The statistical analysis was performed by Prism (GraphPad Software, La Jolla, CA). The correlation between the genera and EAE clinical scores was performed by using Spearman's rank correlation coefficient. Statistical significance was defined as *P* < 0.05.

## Results

### *L. reuteri* Suppresses the Development of EAE in Mice

To determine whether *L. reuteri* treatment can modulate the progression of EAE, we treated mice with 10^8^ CFU/day of *L. reuteri* from 0 to 20 days after MOG immunization (Figure 1A). The clinical scores were significantly suppressed in EAE mice with *L. reuteri* treatment, compared to EAE mice without *L. reuteri* treatment, starting at day 10 post-immunization and lasting until day 20 (Figure 1B) (Table 1). We calculated the incidence of EAE based on the time point at which the mice developed clinical scores of ≥ 0.5. *L. reuteri* significantly reduced the incidence of EAE on each day post-immunization (Table 1). In addition, inflammatory cell infiltration was examined in the spinal cord. H&E staining showed that EAE mice had more inflammatory cells in the spinal cord compared with control mice, and *L. reuteri* treatment consistently reduced inflammatory cell infiltration EAE mice (Figures 1C,D). Immunohistochemical staining indicated that T cells (CD3^+^) were increased in EAE spinal cord tissues when compared to normal mice, while CD3^+^ cell staining was significantly decreased in *L. reuteri*-treated EAE mice (Figures 1C,E). Macrophages (CD68^+^) were increased in spinal cords of EAE mice when compared to normal mice, but spinal cord CD68^+^ cells were moderately decreased by *L. reuteri* treatment (Figures 1C,F). Altogether, these results demonstrate that *L. reuteri* treatment ameliorates the severity of EAE in mice by reducing inflammatory cell infiltration in the spinal cord.

**Table 1:** Clinical assessments of EAE mice affected by *Lactobacillus reuteri*.

Assessments^*^		**Days Post-immunization**											
	**Group**	**d10**	**d11**	**d12**	**d13**	**d14**	**d15**	**d16**	**d17**	**d18**	**d19**	**d20**
Incidence (%)^**^	Ctrl	0	0	0	0	0	0	0	0	0	0	0
	EAE	27.5	52.5	80	87.5	87.5	97.5	97.5	100	100	100	100
	EAE+LR	10	20	42.5	62.5	72.5	80	80	80	85	85	85
Maximal clinical scores	Ctrl	0	0	0	0	0	0	0	0	0	0	0
	EAE	1.5	3	3.5	3.5	4	4	4	4	4.5	4.5	4.5
	EAE+LR	0.5	2	3	3.5	3.5	4	4	4	4	4	4
Cumulative average clinical scores^***^	Ctrl	0	0	0	0	0	0	0	0	0	0	0
	EAE	0.2	0.6	1.1	1.9	2.1	2.5	2.9	2.9	3	3	2.8
	EAE+LR	0.07	0.2	0.5	0.9	1.2	1.7	2.0	2.1	2.2	2.2	2.0

* *Assessments summarized from 4 independent experiments of three groups of Ctrl, EAE, and EAE+LR. Total animal numbers N = 39 in Ctrl group, N = 40 in EAE group and N = 37 in EAE+LR group*.

** *Calculation of the incidence of EAE based on once the mice had clinical scores of ≥ 0.5 defined as EAE. P < 0.05 or < 0.01 between groups of EAE and EAE+LR at each day*.

*** *p < 0.01 or p < 0.001 between groups of EAE and EAE+LR at each day (see Figure 1B)*.

### *L. reuteri* Decreases T_H_1/T_H_17 Cells and Their Associated Cytokines and Reduces MOG_35−55_-Stimulated Cell Proliferation in EAE Mice

**Figure 2:**
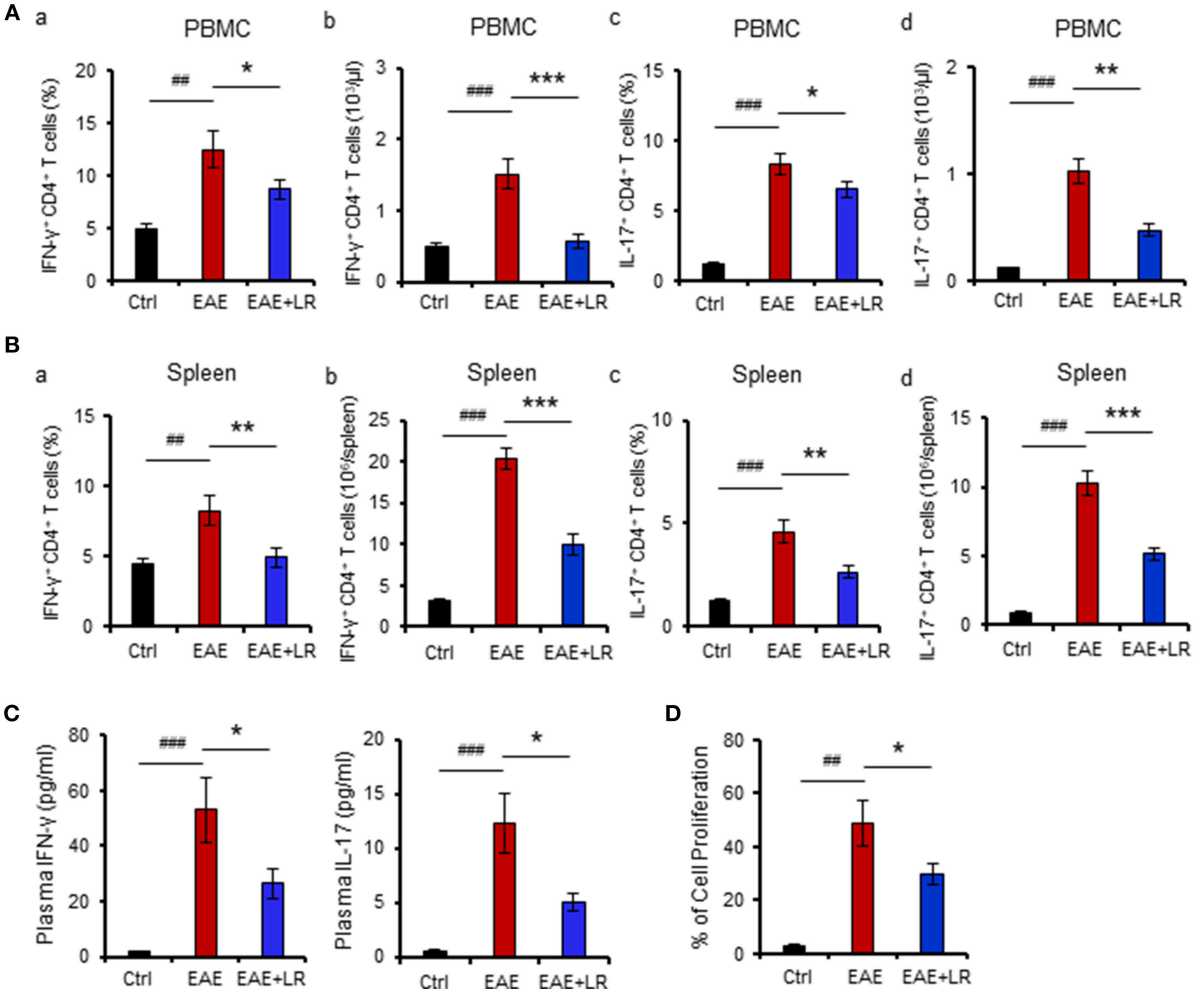
*L. reuteri* treatment decreases T_H_1/T_H_17 cells and their associated cytokines, and reduces MOG_35−55_-stimulated cell proliferation in EAE mice. **(A)** PBMCs. **(a)** the frequency of IFN-γ^+^ CD4^+^ T cells (T_H_1), **(b)** the absolute number of IFN-γ^+^ CD4^+^ T cells, **(c)** the frequency of IL-17^+^ CD4^+^ T cells (T_H_17), and **(d)** the absolute number of IL-17^+^ CD4^+^ T cells. **(B)** Spleen. **(a)** The frequency of IFN-γ^+^ CD4^+^ T cells, **(b)** the absolute number of IFN-γ^+^ CD4^+^ T cells, **(c)** the frequency of IL-17^+^ CD4^+^ T cells, and **(d)** the absolute number of IL-17^+^ CD4^+^ T cells. **(C)** Plasma IL-17 and IFN-γ levels. **(D)** The percentage of cell proliferation of splenocytes isolated from the mice at d12 post-immunization responded to *in vitro* MOG_35−55_ stimulation (see Materials and Methods) in Ctrl, EAE, and EAE+LR mice (*n* = 10 mice per group). Data are presented as mean ± SEM. *In vitro* stimulation assays of PBMCs and splenocytes were performed in triplicate. ^*^p < 0.05, ^**^p < 0.01,^***^p < 0.001. EAE+LR vs. EAE. ^##^*p* < 0.01, ^###^*p* < 0.001. EAE vs. Ctrl.

The immunopathology of MS appears to be mediated mainly by T_H_17 cells but also involves T_H_1 cells (4). We next examined whether *L. reuteri* treatment can alter the composition of lymphocytes and their associated cytokines. Flow cytometric analysis demonstrated that the proportions of T_H_1 and T_H_17 cells among PBMCs and splenocytes were increased in EAE mice. Both the percentage (Figures 2Aa,c,Ba,c) and absolute numbers (Figures 2Ab,d,Bb,d) of T_H_1 and T_H_17 cells were increased in EAE but were reduced after *L. reuteri* treatment (Figures 2A,B). In addition, *L. reuteri* treatment significantly reduced the plasma levels of pro-inflammatory cytokines IL-17 and IFN-γ in mice with EAE (Figure 2C). These data indicate that *L. reuteri* treatment not only decreases the proportion of circulating T_H_1 and T_H_17 cells in EAE but also reduces circulating levels of their associated cytokines. In addition, we observed that splenocytes isolated from the mice after 12 day post-immunization with MOG_35−55_ had increased cell proliferation *in vitro*, which was decreased by *L. reuteri* treatment (Figure 2D).

### *L. reuteri* Remodels EAE-Associated Intestinal Microbiota in Mice

**Figure 3:**
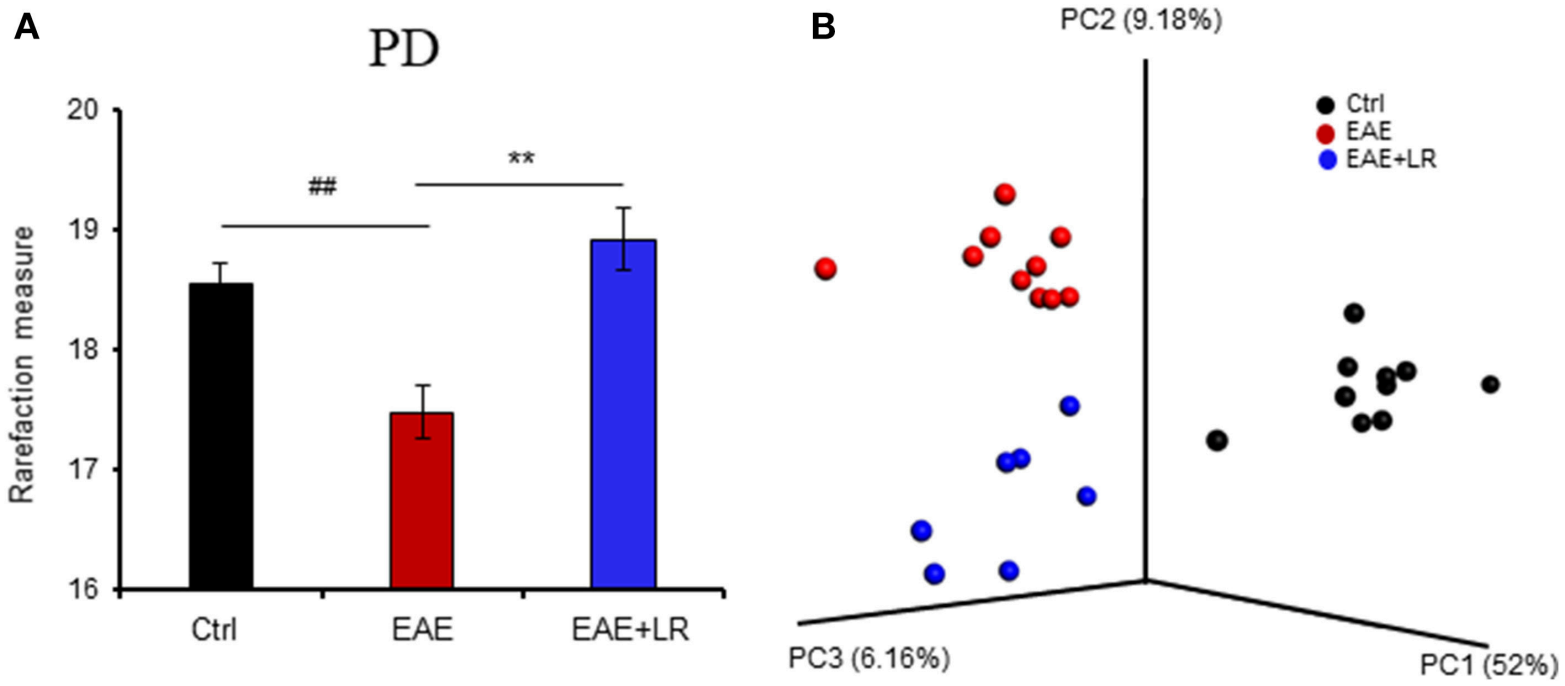
*L. reuteri* treatment modulates the diversity of the gut microbiota. **(A)** Gut microbial Phylogenetic Diversity (PD) whole tree analysis, comparing groups of Ctrl, EAE, and EAE+LR mice (*n* = 7–10 mice per group). **(B)** Unweighted UniFrac-based 3D PCoA analysis of gut microbiota of Ctrl, EAE, and EAE+LR mice (*n* = 7–10 mice per group). Data are presented as mean ± SEM. ^**^p < 0.01. EAE+LR vs. EAE. ^##^*p* < 0.01. EAE vs. Ctrl.

To determine the microbial populations of colonic contents that exhibited significant differences comparing Ctrl, EAE, and EAE+LR groups, we surveyed fecal bacterial populations by 16s rRNA gene sequencing. The gut microbiota of EAE mice was characterized by less alpha diversity than that of Ctrl mice when measured with PD whole tree; however, other alpha diversity metrics (Chao1, Observed Species, Shannon, and Simpsons) uniformly failed to show any difference in alpha diversity (Figure 3A). Unweighted UniFrac-based 3D principal coordinate analysis (PCoA) revealed a strong effect of *L. reuteri* on the beta diversity of the gut microbiota in EAE mice (Figure 3B). Remarkably, EAE+LR samples clustered distinctly from Ctrl or EAE samples (Figure 3B), indicating robust differences in the membership of gut bacteria between Ctrl, EAE, and EAE+LR mice.

**Figure 4:**
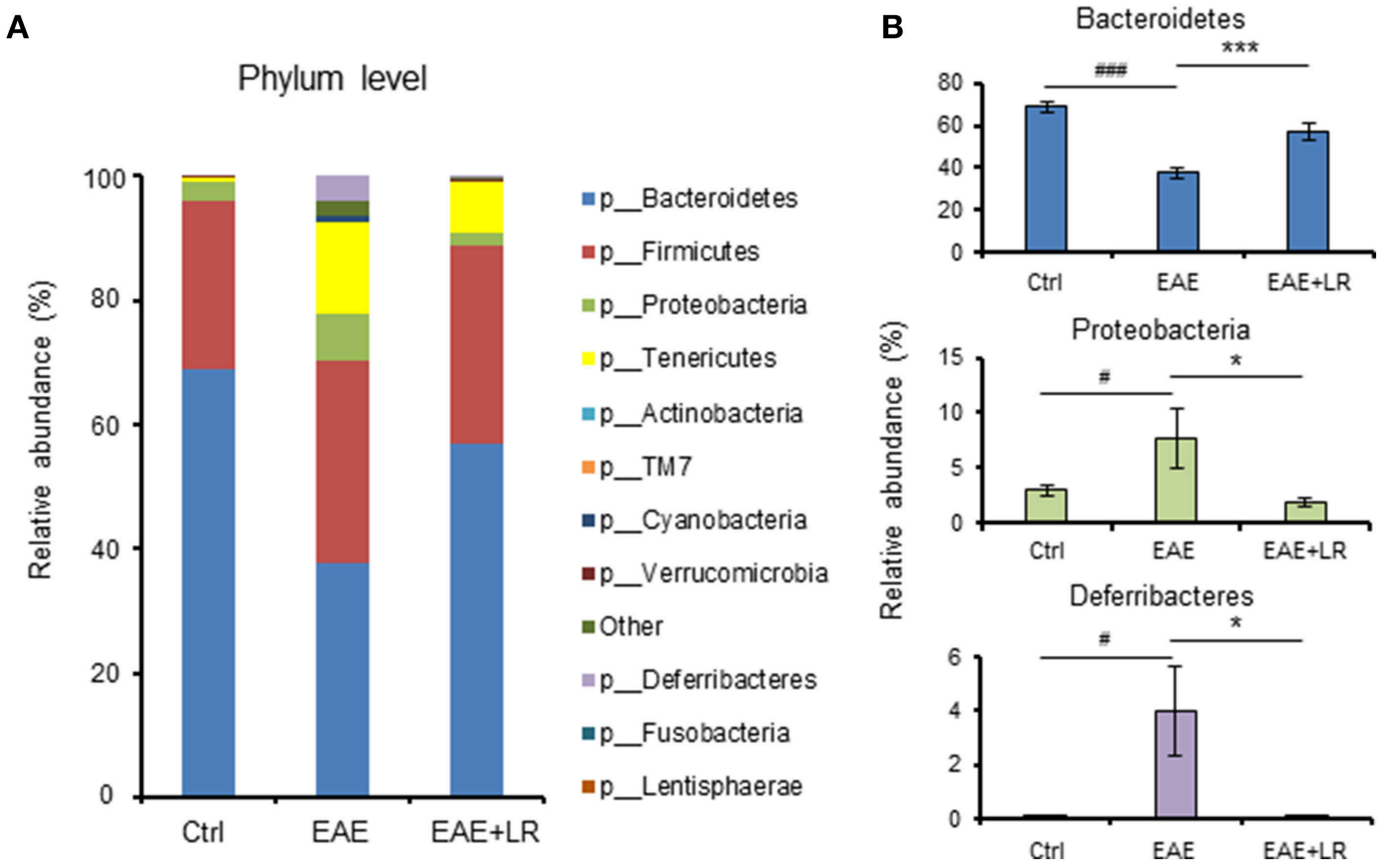
*L. reuteri* treatment remodels EAE-associated intestinal microbiota at the phylum level. **(A)** Relative abundance of bacteria at the phylum level for Ctrl, EAE, and EAE+LR mice (*n* = 7–10 mice per group). **(B)** Relative abundance of *Bacteroidetes, Proteobacteria*, and *Deferribacteres* at the phylum level for Ctrl, EAE, and EAE+LR mice (*n* = 7–10 mice per group). Data are presented as mean ± SEM. ^*^p < 0.05, ^***^p < 0.001. EAE+LR vs. EAE. ^#^*p* < 0.05, ^###^*p* < 0.001. EAE vs. Ctrl.

Taxonomy-based analysis of gut microbiota indicated that the gut microbiota in stool samples from Ctrl, EAE, and EAE+LR mice included four major phyla, *Firmicutes, Bacteroidetes, Proteobacteria*, and *Tenericutes* (Figure 4A). The relative abundances of *Proteobacteria* and *Deferribacteres* were significantly increased in the colonic contents of EAE mice, while the relative abundance of *Bacteroidetes* was reduced. *L. reuteri* treatment reversed the effects of EAE on the relative abundance of these phyla in EAE mice (Figures 4A,B).

**Figure 5:**
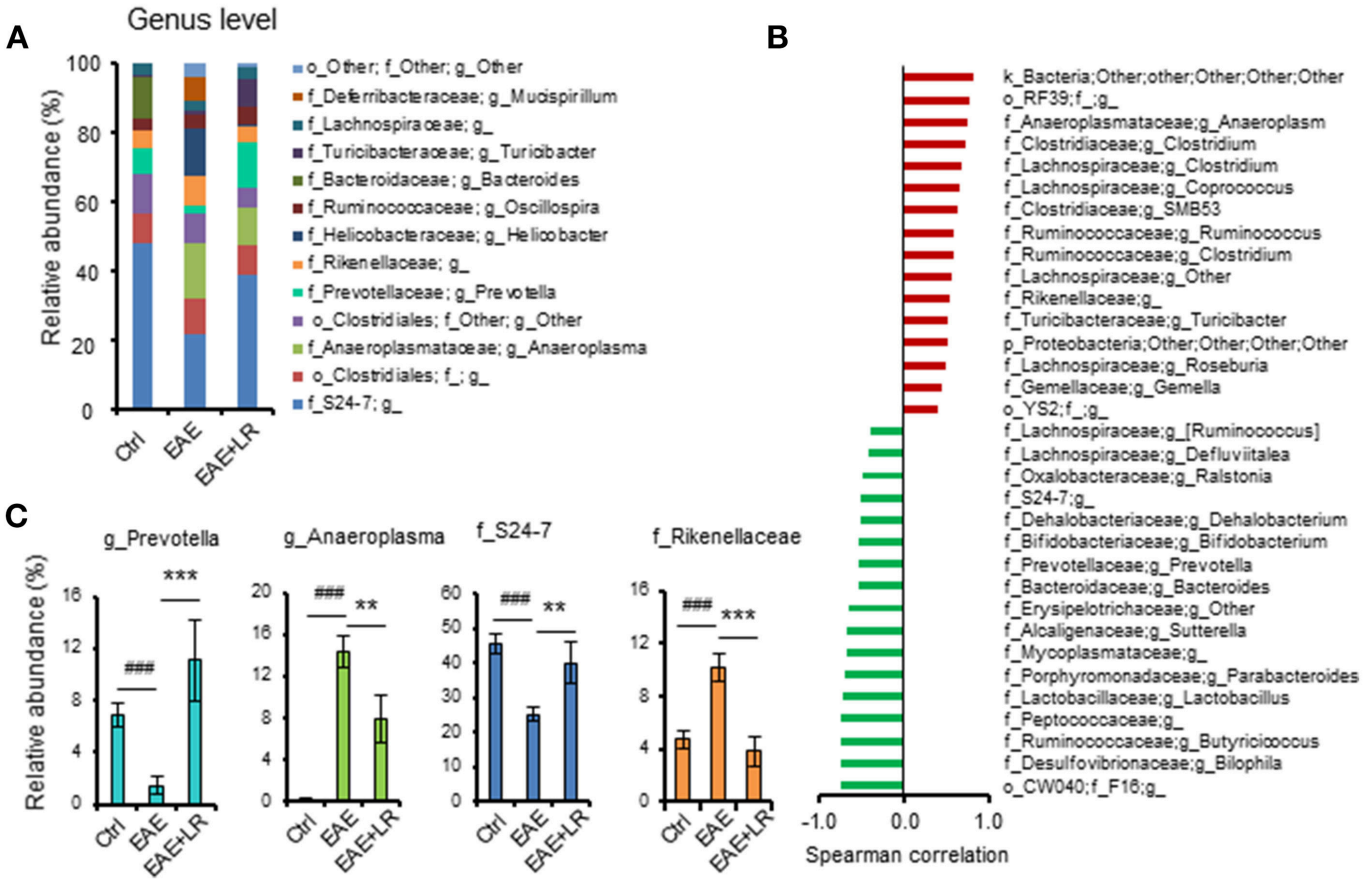
*L. reuteri* treatment remodels EAE-associated intestinal microbiota at the Genus level. **(A)** Relative abundance of predominant bacteria (> 1% of total bacteria) at the genus level for Ctrl, EAE, and EAE+LR mice (*n* = 7–10 mice per group). **(B)** The Spearman correlation between gut microbiota (o, order; f, family; g, genus) and the EAE clinical scores of all mice. **(C)** Relative abundance of *Prevotella, Anaeroplasma, S24-7*, and *Rikenellaceae* were compared among the mice of Ctrl, EAE, and EAE+LR groups, respectively, (*n* = 7–10 mice per group). Data are presented as mean ± SEM. ^**^p < 0.01, ^***^p < 0.001. EAE+LR vs. EAE. ^###^*p* < 0.001. EAE vs. Ctrl.

According to our analysis of predominant bacteria (>1% of total bacteria) at the genus level, the relative abundance of *Anaeroplasma* and *Rikenellaceae* was increased, while the relative abundance of *Prevotella* and *S24-7* was reduced in EAE mice. After oral feeding of *L. reuteri* to EAE mice, the percentages of these genera in gut microbiota were improved by *L. reuteri* treatment (Figures 5A,C).

To explore the relationship between gut microbiota and the severity of EAE, we used the Spearman correlation to compare the composition of gut microbiota at the genus level with the clinical scores (Figure 5B). Results showed that 16 genera had a positive correlation (*p* < 0.05) with clinical scores, while 17 genera had a negative correlation (*p* < 0.05). Interestingly, *Bifidobacterium, Prevotella, Lactobacillus*, and *S24-7* had a negative correlation, indicating improved clinical scores. Conversely, the genera *Clostridium, Anaeroplasma, Ruminococcus*, and *Rikenellaceae* positively correlated with more severe clinical scores. *L. reuteri* changed the relative abundance of certain microbial taxa relevant to the clinical scores in EAE mice (Figures 5A,C). Altogether, these results indicated that EAE-associated gut microbial dysbiosis could be reprogrammed by oral administration of *L. reuteri* treatment.

### Predictive Model Using Random Forests

**Figure 6:**
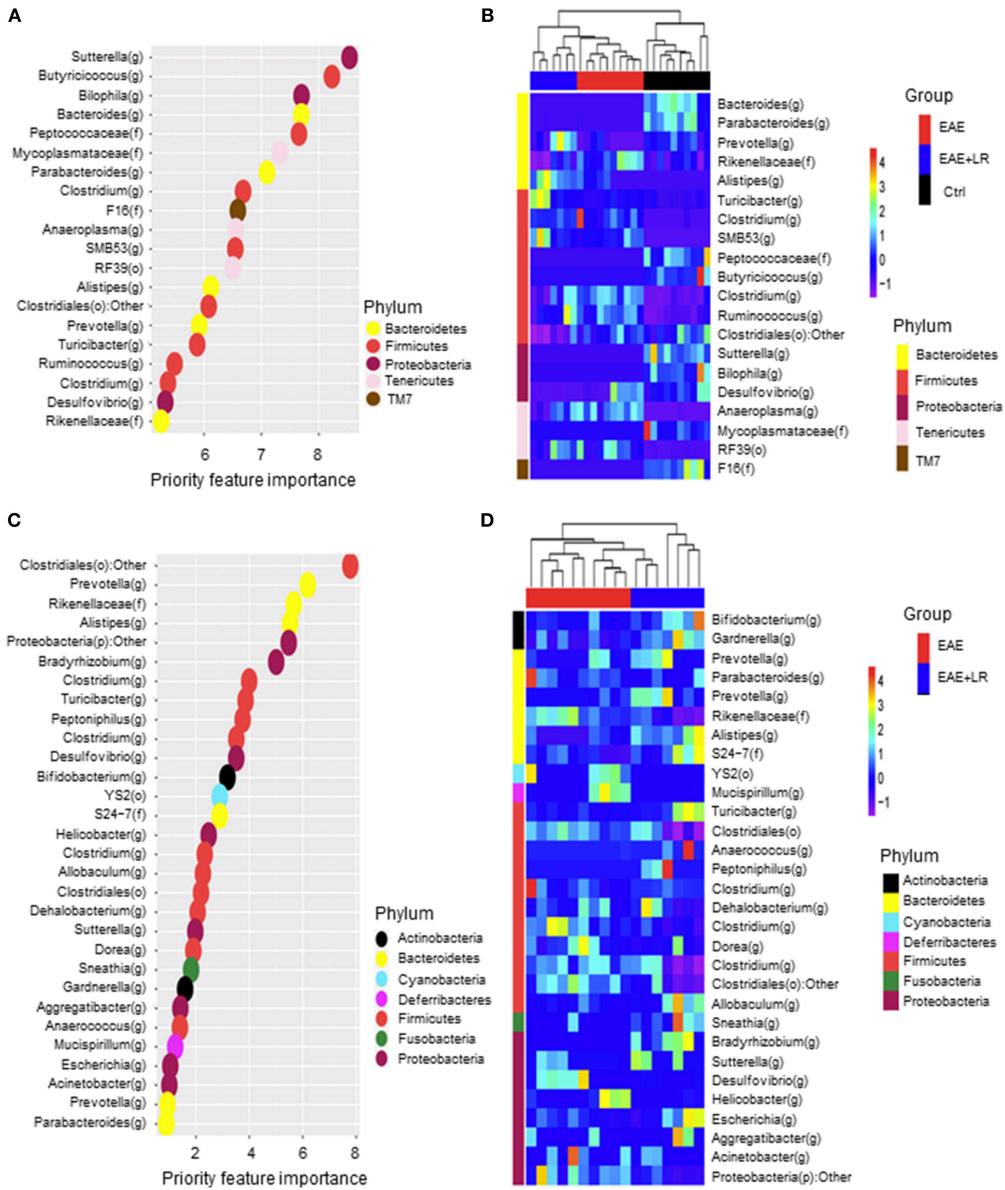
Predictive model based on the relative abundance profile at the genus level using Random Forest analysis. **(A)** Predictive power of individual genera (Top 20 most important bacteria at genus level) with three group (Ctrl, EAE, and EAE+LR) comparisons assessed by Random Forest (RF) analysis (o, order; f, family; g, genus). **(B)** Heatmap based on the relative abundance of bacteria from **(A)** top 20 most important bacteria at the genus level of Ctrl, EAE, and EAE+LR mice. **(C)** Predictive power of individual genera (Top 30 most important bacteria at genus level) with two group (EAE and EAE+LR) comparisons assessed by RF analysis. **(D)** Heatmap based on the relative abundance of bacteria from **(C)** top 30 most important bacteria at the genus level of EAE and EAE+LR mice. Hierarchical clustering shows that Ctrl, EAE, or EAE+LR samples tend to cluster together, respectively.

We used Random Forests (RF) to build an EAE-predictive model by using the genus-level relative abundance data. The relative importance of each genus in the predictive model was assessed using mean decreasing accuracy. We selected 20 significant genera as the signature gut microbiota to compare with groups Ctrl, EAE, and EAE+LR (Figure 6A). Eight genera came from the phylum *Firmicutes* and the rest came from the phyla *Bacteroidetes, Tenericutes*, or *Proteobacteria*. To test whether these 20 genera had sufficient EAE-predictive power in our sample sets, we performed hierarchical clustering based on the relative abundance of these 20 genera. Results showed that the samples from Ctrl, EAE, or EAE+LR groups clustered together, respectively (Figure 6B). Additionally, we also selected 30 significant genera as the signature gut microbiota to compare with the groups of EAE and EAE+LR treatment, which predicts importance of signature microbiota changed by the disease or by *L. reuteri* (Figure 6C). Eleven genera came from the phylum *Firmicutes*, and the rest came mainly from the phyla *Proteobacteria* and *Bacteroidetes*. This predictive model confirmed that certain genera had a significant correlation with the clinical scores and were predictive of EAE. For example, *Sutterella, Bacteroides, Parabacteroides, Prevotella* were all decreased in EAE, whereas *Rickenellaceae, Ruminococcus, Anaeroplasma* were increased in EAE (Figure 6D). This predictive model additionally confirmed that certain genera were predictive of *L. reuteri* treatment modifications in EAE, including *Prevotella, Rickenellaceae, and Anaeroplasma*.

## Discussion

Our current study demonstrates that mice with EAE have a distinct gut microbiota compared to normal mice. Certain gut microbes showed positive or negative correlations with the clinical EAE scores. Probiotic *L. reuteri* treatment of EAE mice modified the relative abundance of EAE-associated microbiota, reducing the clinical symptoms and inflammation during the EAE development. Probiotics have beneficial effects on the host by regulating the intestinal microbial communities and modulating of inflammatory immune responses—both locally in the gut and systemically. Early administration of *L. reuteri* reduces pathogen colonization (24) and has the potential to reduce the risk of necrotizing enterocolitis (NEC) in infants (25, 26). In animal models of NEC, we have demonstrated that *L. reuteri* reduces incidence and severity of NEC via modulation of Toll-like receptor-4 (TLR4) and nuclear factor kappa-light-chain-enhancer of activated B cells (NF-kB) signaling in the intestine (27). *L. reuteri* coordinately modulates inflammatory effector T cells counterbalanced by Foxp3^+^ Treg cells in the intestinal mucosa during NEC, a beneficial effect which is mediated by TLR2 (16, 28, 29).

*L. reuteri* prolongs the survival of mice suffering from Foxp3^+^ Treg-deficiency-induced autoimmune disease (called the scurfy mouse). We have extensively studied this model of a disease characterized by immune dysregulation, polyendocrinopathy, and enteropathy with X-linked inheritance (IPEX syndrome) that is seen in humans (13). We recently identified a novel mechanism by which this probiotic and its metabolite inosine act on an adenosine receptor expressed on inflammatory T cell to inhibit inflammation (13, 30). Because we observed that the *L. reuteri* strain 17938 inhibits the differentiations of naïve CD4^+^ T cells into T_H_1, T_H_2 (13), and T_H_17 cells *in vitro* (data not shown), we decided to “put this strain to the test” in the EAE model of MS, a condition mediated primarily by T_H_1 and T_H_17 cells (31).

We began orally feeding *L. reuteri* by gavage simultaneously with the first inoculation of MOG_35−55_ (d0), and we gave the probiotic until the end of the experiment at d20. Mouse EAE symptoms were visible as early as d10. Oral administration of *L. reuteri* significantly reduced the incidence of EAE and EAE severity scores starting on d10 until d20 post-immunization. Previous studies of probiotics given for EAE used different regimens for prophylaxis. Various authors gave a probiotic daily beginning 3 weeks before (32, 33), 12 days before (34), 7 days before (35–37) or 0 days before the first inoculation of MOG_35−55_ (that is, on d0) (38). For other MS treatments (medications), study groups have administered preventative treatment on d0 (39–41) or d2-post-immunization (42). Our protocol of simultaneous oral administration at the same time as with MOG_35−55_ sensitization in this study falls into the prophylactic window. For therapeutic regimens, compounds or probiotics have been administered starting at d11 or d20 post-immunization (39, 42).

Previous studies showed that the effects of probiotic Lactobacilli on EAE autoimmunity are strain-, and EAE model-dependent (43, 44). *Lactobacillus casei* strain Shirota (LcS) has been reported to be associated with increases in T_H_1-associated cytokines in a Lewis rat EAE model (35), which raised concerns about the safety of this strain in MS patients. Further studies evaluated the safety of *Lactobacillus casei* strain Shirota (LcS) together with *Bifidobacterium breve* strain Yakult (BbY) when given to Lewis rats with EAE. However, in this series of experiments investigators found that neither LcS nor SbY exacerbates EAE (45). Another study demonstrated that even though LcS upregulated peripheral IL-17 responses; it did not exacerbate neurological symptoms in EAE (37). Another probiotic mixture, IRT5, representing a combination of five probiotic strains, *Streptococcus thermophilus, L. reuteri, Bifidobacterium bifidium, Lactobacillus acidophilus*, and *Lactobacillus casei*, was given as pretreatment 3 weeks before disease induction. Investigators found that treatment with IRT5 during induction of EAE delayed the disease onset (32). Previous studies demonstrated that *Bifidobacterium animalis* PTCC1631 in combination with *L. plantarum* A7 also ameliorated neuroinflammation in the EAE mouse model (38).

Recently, investigators using this MS model tested different probiotic strains and selected mixtures, including *Lactobacillus crispatus* LMG P-23257, *Lactobacillus rhamnosus* ATCC 53103, *Bifidobacterium animalis* subspecies Lactis BB12®, and *Bifidobacterium animalis* subspecies Lactis LMG S-28195. Their results indicated that selective probiotic mixtures effectively modulate disease symptoms in the EAE model (36). Administration of the selected mixtures altered CD4^+^ T cell subset balance, inhibiting the pro-inflammatory T_H_1/T_H_17 polarization while inducing IL10-producing Foxp3^+^ Treg cells (36, 38). In our study, *L. reuteri* reduced the inflammatory infiltration in the spinal cord, especially invading T cells (CD3^+^) and macrophages (CD68^+^), reducing IFN-γ-producing T_H_1 and IL-17-producing T_H_17 cells. However, we did not observe a difference with respect to the levels of circulating IL4-, and IL-10-producing T cells (and their associated cytokines IL-4 and IL-10 in plasma (data not shown). This finding indicates that strain *L. reuteri* DSM 17938 specifically acts on T_H_1 and T_H_17 subsets in the EAE model.

Other studies in the EAE model which tested a single probiotic strain, *Lactobacillus helveticus* SBT2171 (33) or another combination probiotic *Enterococcus faecium* strain L-3 (46) with *Prevotella histicola* (47) showed differential modulation of immune cells and amelioration of EAE. Gut microbes and their products therefore must participate as key participants in the development of MS. Germ-free mice are fully protected from spontaneous EAE (6), while gut microbiota from MS patients when transferred facilitates the spontaneous development of EAE in mice (48). As final evidence, antibiotics can alter the severity of MS (10, 11).

In the current study, we analyzed the changes of gut microbiota derived from colonic contents, revealing significant differences among the groups in the beta diversity distance matrix (PCoA). For alpha-diversity, among five metrics (Chao1, Observed Species, PD whole tree, Shannon, and Simpson), only the PD whole tree analysis showed significantly differences. At the phylum level, *L. reuteri* treated EAE mice had restoration toward normal relative abundance of *Bacteroidetes*, but continued to show reduced relative abundances of *Proteobacteria* and *Deferribacteres*. We conclude that *L. reuteri* promotes the growth of beneficial commensal microbes (*Bacteroidetes*) and while reducing the abundance of pathobionts (*Proteobacteria*) or potentially pathogenic (*Deferribacteres*) gram-negative organisms.

Recent studies noted that the interaction between *Bacteroidetes* and their animal host is one of mutualism rather than commensalism (49). Mutualism refers to a situation in which both organisms benefit, whereas commensalism refers to a state in which one species benefits but the other is not harmed. *Bacteroidetes* are also beneficial to the normal development of the gastrointestinal tract (GIT), enhancing the immune system and activation of T-cell mediated responses (50). Furthermore, *Bacteroidetes* limit the colonization of the gastrointestinal tract by potential pathogenic bacteria (51). Gut *Bacteroidetes* produce butyrate, facilitate bile acid metabolism, and can transform toxic compounds (52, 53).

At the genus level, several EAE-associated bacteria (*Anaeroplasma* and *Rikenellaceae*) were reduced by *L. reuteri* treatment, at the same time as the low relative abundance of *Prevotella* and S24-7 rebounded back to normal. Human studies of MS patients have also indicated that the relative abundance of *Prevotella* and *Lactobacilli* are decreased compared to healthy controls (7, 8) and these 2 taxa increase after treatment with MS-directed phamacotherapy (7). Interestingly, *Prevotella* in particular has been associated with phytoestrogen metabolism (54–56). Metabolites derived from phytoestrogens play a critical role in producing anti-inflammatory responses (57). Treatment with certain phytoestrogens can suppress and/or protect mice from EAE (58–60). Future studies should investigate the role of *Prevotella-*derived phytoestrogen products in the prevention and therapy of autoimmune disease. In addition, *Prevotella* and *Lactobacillus* can ferment carbohydrates to yield short chain fatty acids (SCFA) which are known for their beneficial immunoregulatory functions (61–63).

After analyzing the correlation between the significantly changed bacteria with clinical EAE scores, we concluded that among all the bacteria, 16 genera bacteria positively correlated with clinical EAE scores, while 17 negatively correlated with clinical EAE scores. Our data therefore further the understanding EAE–associated bacteria and provide additional information that may assist in modulating gut bacteria to change the development of MS. Toward this end, we used predictive models to confirm the bacterial taxa differentially expressed and their importance in the control of the disease. *Prevotella* and *Rikenellaceae* had priority importance to EAE and EAE with LR treatment (Figure 6). They are members of the phylum *Bacteroidetes*, but as shown in Figure 5, *L. reuteri* increased the abundance of *Prevotella* that were decreased in EAE while *L. reuteri* reduced the abundance of *Rikenellaceae*. This is significant because *Prevotella* negatively and *Rikenellaceae* positively correlated with clinical disease severity. Our results provide clear evidence that resetting gut microbiota should be considered as an adjunctive therapeutic strategy for treating MS.

In summary, we have found alterations in the gut microbiota that correlate with changes of neuroinflammation in a mouse model of MS. A novel therapeutic strategy for MS may consist of the use of probiotics, prebiotics, defined microbial communities, or even fecal microbiota transplantation—aiming to remodel the microbiome in this disease.

## Author Contributions

BH, YL, JL, and JR conceived and designed the experiments. BH, TH, JF, SP, and YL performed all experiments and analyzed the data. XT, CT, EB, and ML contributed to microbiota analysis. JL guided EAE mouse model and clinical EAE scoring. MB and JL contributed the spinal cord sample histological processing and analysis. JC and DT assisted flow cytometry and immune cell analysis. BH, YL, XT, CT, MB, JL, DT, and JR wrote the paper and edited the manuscript. All authors read and approved the final manuscript.

### Conflict of Interest Statement

The authors declare that the research was conducted in the absence of any commercial or financial relationships that could be construed as a potential conflict of interest.
